# Targeting the cyclin dependent kinase and retinoblastoma axis overcomes standard of care resistance in BRAF^*V600E*^-mutant melanoma

**DOI:** 10.18632/oncotarget.23649

**Published:** 2017-12-23

**Authors:** Antoneicka L. Harris, Samantha E. Lee, Louis K. Dawson, Laura A. Marlow, Brandy H. Edenfield, William F. Durham, Thomas J. Flotte, Michael Thompson, Daniel L. Small, Aidan J. Synnott, Svetomir N. Markovic, John A. Copland

**Affiliations:** ^1^ Department of Cancer Biology, Mayo Clinic, Jacksonville, FL, USA; ^2^ Charles River Discovery Services, Morrisville, NC, USA; ^3^ Department of Laboratory Medicine and Pathology, Mayo Clinic, Rochester, MN, USA; ^4^ Hematology/Oncology Department, Mayo Clinic, Rochester, MN, USA

**Keywords:** melanoma, patient-derived tumor xenograft (PDTX), cyclin dependent kinase 4/6 (CDK4/6) inhibitors, retinoblastoma (Rb), mutant BRAF

## Abstract

Patient-derived tumor xenograft (PDTX) mouse models were used to discover new therapies for naïve and drug resistant *BRAF*^*V600E*^-mutant melanoma. Tumor histology, oncogenic protein expression, and antitumor activity were comparable between patient and PDTX-matched models thereby validating PDTXs as predictive preclinical models of therapeutic response in patients. PDTX models responsive and non-responsive to BRAF/MEK standard of care (SOC) therapy were used to identify efficacious combination therapies. One such combination includes a CDK4/6 inhibitor that blocks cell cycle progression. The rationale for this is that the retinoblastoma protein (pRb) is 95% wildtype in *BRAF* mutant melanoma. We discovered that 77/77 stage IV metastatic melanoma tissues were positive for inactive phosphorylated pRb (pRb-Ser780). Rb is hyperphosphorylated and inactivated by CDK4/6:cyclin D1 and when restored to its hypophosphorylated active form blocks cell cycle progression. The addition of a CDK4/6 inhibitor to SOC therapy was superior to SOC. Importantly, triple therapy in an upfront treatment and salvage therapy setting provided sustained durable response. We also showed that CDK4/6 blockade resensitized drug resistant melanoma to SOC therapy. Durable response was associated with sustained suppression of pRb-Ser780. Thus, reactivation of pRb may prove to be a clinical biomarker of response and the mechanism responsible for durable response. In light of recent clinical trial data using this triple therapy against *BRAF*^*V600E*^-mutant melanoma, our findings demonstrating superior and prolonged durable response in PDTX models portend use of this therapeutic strategy against naïve and SOC resistant *BRAF* ^*V600E*^-mutant metastatic melanoma coupled with pRB-Ser780 as a biomarker of response.

## INTRODUCTION

Metastatic melanoma is a disease with poor prognosis [1], primarily due to its complex tumor heterogeneity, distinct profiles of somatic mutations involved in tumorigenesis [2], and intrinsic resistance to both chemotherapy and radiotherapy [3]. Chromosomal alterations, such as *BRAF* mutations [4] lead to constitutive activation of the mitogen-activated protein kinase (MAPK) pathway, a prominent signaling pathway in human metastatic melanoma [5]. The recent development of targeted therapies (e.g. vemurafenib, dabrafenib, and trametinib) and immune therapies (e.g. ipilimumab, pembrolizumab, and nivolumab) has improved disease outcomes. For example, single agent BRAF inhibition has increased overall survival (OS) by 20% [6] and immune therapies, such as ipilimumab, have increased OS by 32%, with response rates varying between 32-45% [7, 8]. However, despite these improvements, varying mechanisms of resistance occur in patients. For instance, immune therapies are associated with innate resistance, whereas targeted therapies are associated with acquired resistance [9], which the latter is often associated with reactivation of the MAPK pathway which promotes cell proliferation, drug resistance and protection from apoptosis [10]. This underscores the need to identify new therapies that improve disease management and patient survival that suppress cell cycle progression, prevent or reverse drug resistance and promote cell death.

A primary cause of marginal advancements of new agents in oncology is due to lack of preclinical models that recapitulate patient tumor heterogeneity [11]. The complex genetic alterations involved in metastatic melanoma progression require preclinical models to better understand its biology to conceptualize novel combination therapies [12] leading to drug efficacy in humans. PDTX models are considered reliable preclinical models due to their ability to predict clinical activity, mimic patient response to therapy [13], maintain key genes and global pathway activity as that of patients’ tumors [14], possess tumor heterogeneity, and investigate novel therapeutic compounds. We developed *BRAF*^*V600E*^-mutant PDTX mouse models using immune deficient athymic nude mice bearing subcutaneous human metastatic melanoma tissues from patients with distinct clinical treatment response backgrounds.

MAPK pathway activation and cell cycle dysregulation are general hallmarks of melanoma [15] resulting from aberrations in cell proliferation [16], deficiency of the retinoblastoma protein (pRb) [17], mutations in CDK4 [17, 18], and overexpression of cyclin D1 following resistance to BRAF inhibition [19, 20]. In addition, driver mutations in BRAF promote CDK4/6 activation [21], suggesting that *BRAF* mutant cells may be sensitive to anti-CDK4/6 therapy. Secondary mutations in MEK [22] and activation of downstream MEK1 [23] following escape from single agent BRAF inhibition led to the investigation of the current SOC therapy for *BRAF*^*V600E/K*^-mutant melanoma, dual dabrafenib and trametinib treatment. Unfortunately, this therapeutic combination still proves insufficient in escaping drug resistance [24]. In our studies, we investigated the antitumor activity of BRAF, MEK and CDK4/6 inhibitors in combination using both treatment responsive and drug-resistant *BRAF*^*V600E*^-mutant metastatic melanoma PDTXs. We hypothesized that the addition of a CDK4/6 inhibitor to SOC treatment would provide superior antigrowth activity compared to SOC by blocking cell cycle progression through inhibition of pRb phosphorylation. In this study, we report a preclinical strategy to assess tumor sensitivity in *BRAF*^V600E^-mutant melanoma PDTX mouse models to an anti-cancer drug combination determined by oncogenic profiling, molecular analyses, and protein expression. This study identifies targeting the CDK/Rb axis combined with SOC to promote enhanced antitumor activity and tumor regression, and importantly, prolonged therapeutic response while on triple therapy superior to SOC. Moreover, triple therapy overcomes SOC drug resistance.

## RESULTS

### Human tumors express targetable proteins implicating therapeutic benefit from triple therapy

**Figure 1:**
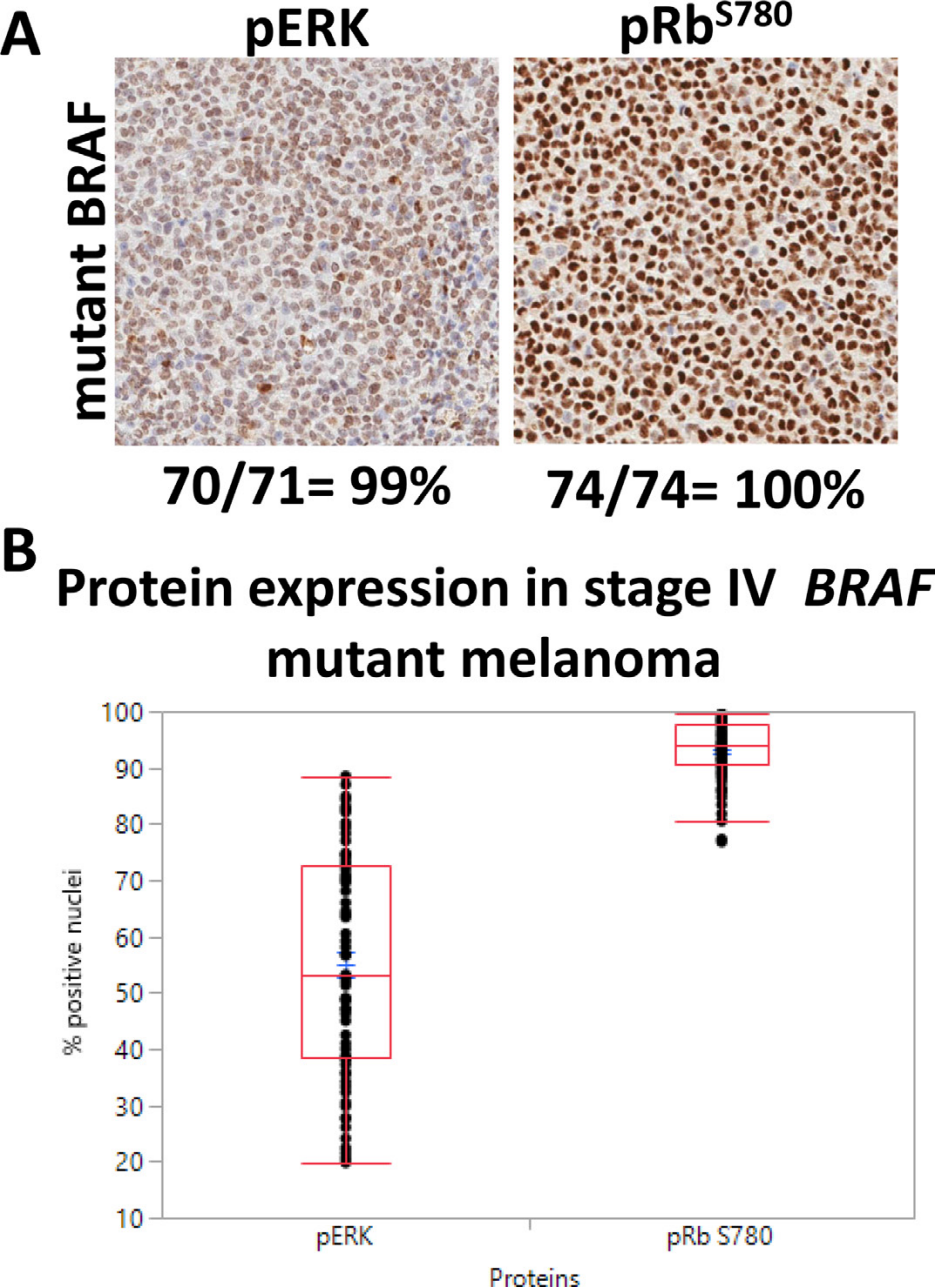
Human tumors confirm pERK and pRb-Ser780 protein expression (**A**) 77 tumor cores from patients with *BRAF* mutant stage IV disease (with or without prior therapy) were analyzed for pERK and pRB-Ser780 protein expression. Representative images are shown for each protein type, along with the total percent of patients who had nuclear protein expression. Tumor cores with insufficient and/or necrotic tissues were excluded. Magnifications are 20×. (**B**) The range in nuclear expression present in the nuclei of each individual patient tumor sample was analyzed, per protein, and compared between samples.

The aberrant activation of ERK (pERK) and inactivation of pRb (phosphorylated Rb-Ser780 or pRb-Ser780) contribute to constitutive oncogenic signaling within tumor cells, which has been previously reported in *BRAF*^*V600E*^-mutant melanoma [25, 26].To confirm the aberrant expression of these two pathways, we examined clinical samples using a TMA of human stage IV melanoma tissues for pERK and pRB-Ser780. Protein expression was analyzed via immunohistochemistry (IHC) with the expectation that these proteins would be present and elevated. Within the TMA, 77 patients were identified as having BRAF mutant disease using a BRAF^V600E^ antibody for IHC. Tumors were regarded positive for protein expression when at least 20% of the nuclei stained positive. Representative images per protein are shown (Figure 1A). Human tumors were 99% positive for pERK, and 100% positive for pRb-Ser780 (Figure 1A). Nuclear protein expression per patient tumor core was present at varying levels. Mean nuclear expression ranged between 55–93% of cells staining positive (Figure 1B). Thus, we confirmed in clinical samples that MAPK and cell cycle proteins that promote tumor proliferation are highly active in stage IV melanoma.

### Histological and genetic comparison showed PDTX matched parental patient tumor tissue

**Figure 2:**
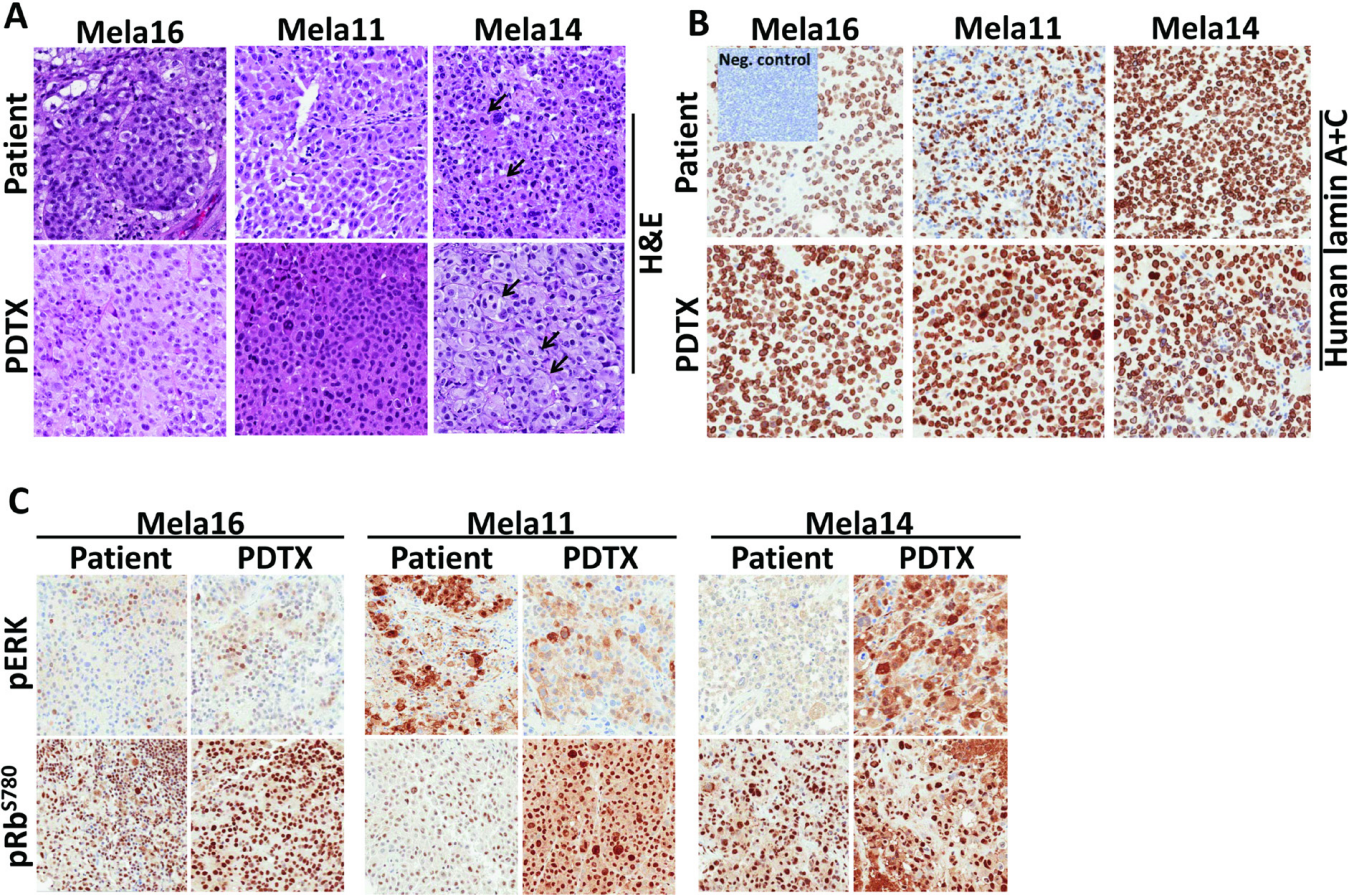
Phenotypic and genetic comparison showed PDTX matched parental patient tumor tissue (**A**)TMAs were constructed to compare and analyze similarities between patient and matched PDTX tumor tissues for tumor architecture via H&E stain, (**B**) human specificity via lamin A+C stain (non-stained cells shown in the patient Mela11 model are non-neoplastic lymphocytes), and (**C**) oncogenic proteins. a, Magnifications for all TMA images are 20×.

The reliability of PDTX mouse models to predict patient response to therapy led us to develop models from patients who were treatment naïve (Mela16), responsive (Mela11) or drug resistant (Mela14) to SOC. All models showed histologic features similar to those of their patient-matched tissue samples (Figure 2A). These features included sheets of melanocytes, which are indicative of a malignant phenotype, and epithelioid cells with abundant eosinophilic cytoplasms and enlarged nuclei. Tumor cells from both Mela16 and Mela11 models were more monomorphic compared to tumor cells from the Mela14 model. An additional cytologic similarity shared between Mela14 patient and its PDTX model is the ability of the tumor cells to form nests (arrows). Human specific Lamin A+C antibody was used as a marker for the identification of human cells in our PDTX mouse models. Its positive expression in the PDTX models confirmed maintenance of human tumor cells. Pancreatic mouse tumor tissue was used as a negative control (Figure 2B). For patient and PDTX comparison of protein expression via IHC, each tumor model had its own distinct protein expression between pERK and pRb-Ser780 (Figure 2C). The overall commonality between each model, both patient and matched-PDTX, was intense nuclear protein expression of pRb-Ser780, revealing an active cell cycle. Collectively, protein expression of pERK and pRb-Ser780 provides evidence that the MAPK and cell cycle pathways are active in metastatic melanoma. For DNA fingerprinting, STR analysis on PDTX tissues showed stable allele sizes for the majority of markers recognized both nationally and internationally as standard for human identification (Table 1). Overall, the STR signature of the PDTX models matched their respective patient tumor tissue. There was evidence of possible genetic drift (asterisks) in Mela11 PDTX at loci D8S1179, Mela14 PDTX at loci D13S158 and Mela16 at loci D7S820 likely due to either loss of heterozygosity or amplification error due to FFPE DNA extract. Matching STR profiles provide evidence that these preclinical models are true melanoma models of patient origin that can be utilized as reliable preclinical models to investigate and develop novel therapies.

**Table 1:** STR profile of patients and PDTXs

	AMEL	D5S818	D13S317	D7S820	VWA	TH01	TPOX	CSF1P0	D18S51	D3S1358	D8S1179	FGA
patient 16	XY	12,13	13,14	8	18,19	8,9.3	11,12	12	14	16,18	12,15	21,22
Mela16 PDTX	XY	12,13	13,14	8, 11	18,19	8,9.3	11,12	12	14	16,18	12,15	21,22
patient 11	XY	11,12	11,12	10	18,19	7,9.3	8	10,12	13,17	16	13,*	21
Mela11 PDTX	XY	11,12	11,12	10	18,19	7,9.3	8	10,12	13,17	16	13,14	21
patient 14	XY	12	11,12	9,11	16,20	9,9.3	8	10,12	10.1,14	*,16	12,14	24,26
Mela14 PDTX	XY	12	11,12	9,11	16,20	9,9.3	8	10,12	10.1,14	15,16	12,14	24,26

STR DNA sequences of both patient and mouse tumors validate genetic identities of developed models. a, Asterisks indicated lost allele.

### Matched PDTX models show common therapeutic responses to that of the matched patient

**Figure 3:**
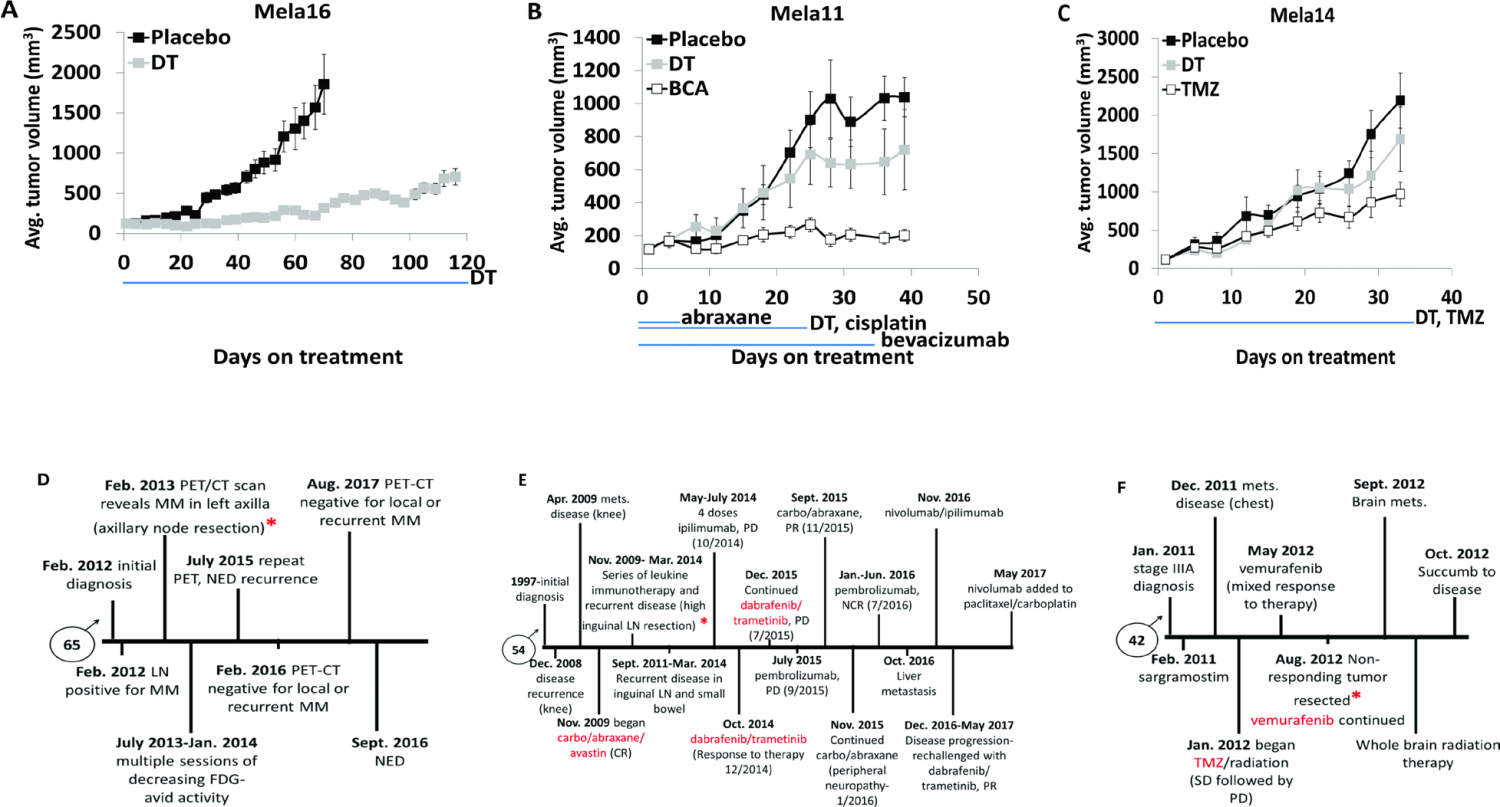
Matched PDTX models show common therapeutic responses to that of the matched patient (**A**–**C**) Mice bearing subcutaneous tumors were dosed as indicated with vehicle (*n* = 10, Mela14 and Mela16; *n* = 8, Mela11), or combination of (25 mg/kg) dabrafenib and (1 mg/kg) trametinib (*n* = 10, Mela14 and Mela16; *n* = 8, Mela11). Both dabrafenib and trametinib were dosed (once daily by mouth). Each *in vivo* model had a distinct response to treatment, ranging in the order of most sensitive to least sensitive (p-values for Mela16, Mela11, and Mela14, are *p* < 0.05, *p* = 0.92, and *p* = 0.97, respectively, when compared to no treatment). (B-C) Mice were also dosed with similar targeted therapy as the respective patient from which the PDTX model was derived. (B) Mice were dosed with the combination of (5 mg/kg) bevacizumab, (8 mg/kg) cisplatin, and (20 mg/kg) abraxane. Bevacizumab was dosed (biwk x 5, ip), cisplatin was dosed (qwk × 3, ip), and abraxane was dosed (qod x 5, iv). (Wilcoxon rank sum test; *p* < 0.05 vs. no treatment). (C) Mice were dosed with (100 mg/kg) TMZ (qd x 5, po). (Wilcoxon rank sum test; *p* = 0.52 vs. no treatment). Red asterisks denotes when tumors were received for development of corresponding PDTX mouse models. The blue line below the x-axis indicates dosing (Rx) period in all studies. The y-axis is mean tumor volume ± SEM. (**D**–**F**) Patient clinical history for disease treatment. A, PDTX drugs were compared to those highlighted in red. b, TMZ, temozolomide; Mets, metastasis; LN, lymph node; Carbo, carboplatin; SLNB, sentinel lymph node biopsy; MM, metastatic melanoma; PET/CT, positron emission tomography-computerized tomography; FDG, fludeoxyglucose; NED, no evidence of disease; DT, dabrafenib+trametinib; BCA, bevacizumab+cisplatin+abraxane; CR, complete response; PD, progressive disease; PR, partial response; NCR, near complete response; SD, stable disease.

We next compared treatment responses between patient and PDTXs to further confirm their capability of predicting human response to treatment when investigating new drugs. We first tested our models with SOC therapy to address the issue that despite the commonality of the driver *BRAF* mutation in melanoma tumors, tumor heterogeneity contributes to different treatment responses. Each PDTX model had a distinct response to combination therapy shown in order of responsiveness (Figure 3A–3C), with Mela14 being completely drug resistant to SOC (Figure 3C). Additional therapies that were relevant to the individual PDTX models were also examined for comparison to the therapy received by the patients. The Mela16 PDTX mouse model was developed from human tumors resected from the Mela16 patient’s axillary nodes (asterisks). However, since the patient was treatment naïve (Figure 3D) no drug-related comparison studies with its matched PDTX mouse model were conducted. As part of a phase II clinical trial, Mela11 patient was initially treated with a triple therapy combination containing avastin (VEGF inhibitor), carboplatin (platinum), and abraxane (taxane) demonstrating a complete response (CR) with no evidence of disease (NED). Ultimately the patient had recurrent disease and was eventually treated with SOC therapy, and after multiple rounds of immune therapy the patient had continued metastatic disease progression (Figure 3E). During one of these sessions of immune therapy, high inguinal lymph node tumors were resected from the patient (asterisks) and collected to create the matched PDTX mouse model. Two of the Mela11 patient treatment regimens (triple and SOC) were conducted in the corresponding PDTX model using bevacizumab, cisplatin, and abraxane (BCA) as the triple clinical trial equivalent. These responses captured the transient responses seen in the patient (Figure 3B).

One year after diagnosis of stage IIIA melanoma, Mela14 patient was treated with the alkylating agent, temozolomide (TMZ), and radiation resulting in stable disease followed by disease progression. Not long after, he was treated with the BRAF inhibitor (vemurafenib) resulting in mixed response to therapy, disease progression and brain metastases (Figure 3F). Tumors that were non-responsive to vemurafenib treatment (asterisks) were resected and used to create the PDTX mouse models. Single agent TMZ treatment resulted in a partial response to therapy in the Mela14 PDTX mouse model (Figure 3C). Treatment with a single BRAF inhibitor (dabrafenib) was performed in the Mela14 PDTX mouse model in a previous experiment and as predicted, there was no response to treatment (Supplementary Figure 1A). Taken together, the similarities in response to therapy between the patient and matched PDTX mouse models confirm their use as viable preclinical tools that predict patient response to therapy.

### Antitumor activity of dabrafenib, trametinib, and palbociclib in combination caused tumor regression and durable response

**Figure 4:**
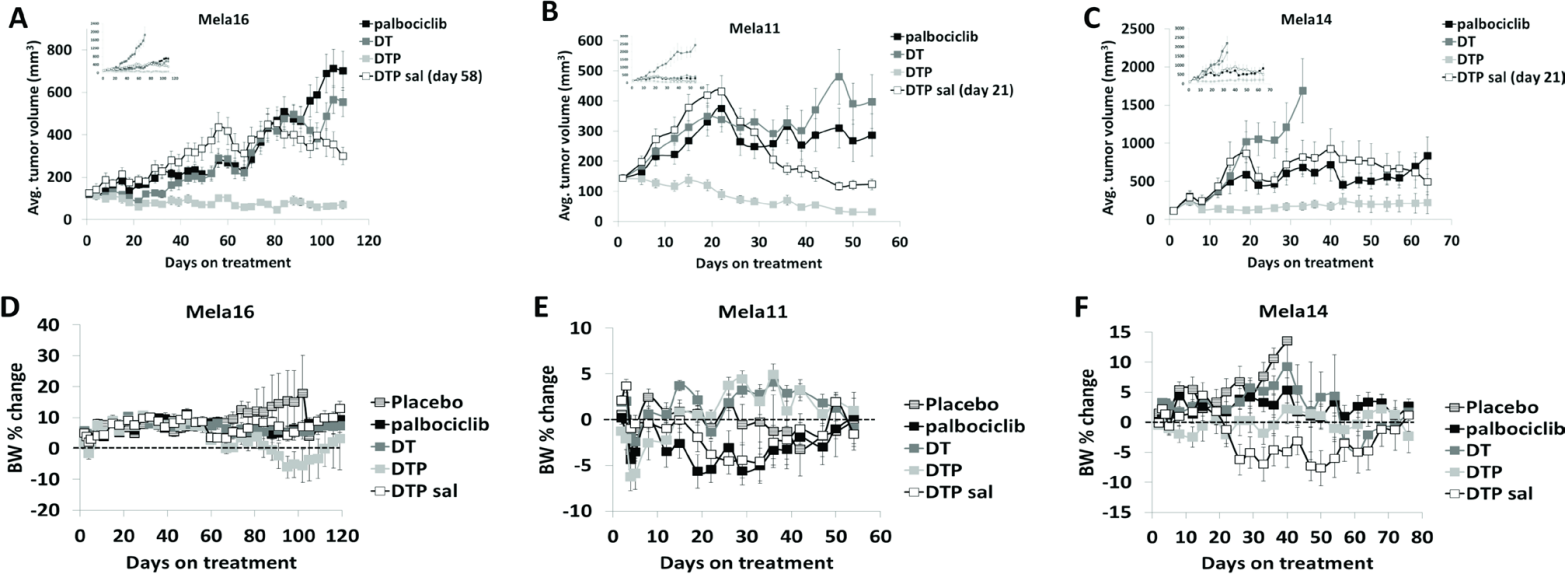
Antitumor activity of dabrafenib, trametinib, and palbociclib in combination **(A–C)** Athymic nude mice bearing subcutaneous tumors were dosed time to endpoint in well-established tumors (∼150 mm^3^). Insets presented in the top left corner include placebo control groups. The y-axis is mean tumor volume ± SEM. With the exception of vehicle versus dabrafenib and trametinib combined therapy in the Mela14 tumors (*p =* 0.46), pairwise comparisons of each treatment versus control are all statistically significant (*p* < 0.05). Salvage therapy was added at day 58 for Mela16 and day 21 for Mela11 and Mela14. Tests between the combination groups and dabrafenib and trametinib combined therapy are also statistically significant (*p* < 0.05). The tests between the combination groups and single agent palbociclib are also statistically significant (*p* < 0.05), with the exception of dual dabrafenib and trametinib treatment in all PDTX models (*p =* 0.23, Mela11; *p =* 0.14, Mela14; *p =* 0.41, Mela16), and DTP salvage in the Mela14 PDTX model (*p =* 0.3). Statistical analyses between DTP and DTP salvage are also statistically significant (*p* < 0.05) with the exception of the Mela14 treated tumors (*p =* 0.07). (**D**–**F**) Change in percent body weight loss was calculated per mouse. The y-axis is change in body weight measured from baseline (day 1) ± SEM. a, DT, dabrafenib + trametinib; DTP, dabrafenib + trametinib + palbociclib; sal, salvage.

Since disruption of the CDK4/Rb pathway is frequent in many melanomas the cyclin D1-CDK4/6-Rb axis is considered a major driver of melanomagenesis [27]. Mechanisms that cause CDK4/6-Rb pathway dysregulation include amplifications in cyclin D1 [28] and activating mutations in CDK4 [29]. Both of these mechanisms can occur as a consequence of acquired BRAF inhibitor drug resistance [19, 20]. Because of this, we investigated the antitumor activity of palbociclib in combination with dual dabrafenib and trametinib treatment (DT) as an upfront triple therapy combination and palbociclib as a salvage therapeutic strategy added to dual SOC treatment when tumors escape dual BRAF/MEK inhibition. Dosing schedule and treatment groups that included SOC, single agent palbociclib, triple therapy and the use of palbociclib as a salvage therapy are described in Table 2. Upfront treatment with the triple therapy combination (DTP) synergistically caused tumor regression with durable response in all three models (Figure 4A–4C; Supplementary Table 1). Treatment with single agent palbociclib elicited antitumor responses similar to SOC treatment in Mela16 and Mela11 (Figure 4A–4B) while also retaining antitumor activity in the SOC resistant Mela14 model (Figure 4C). Palbociclib was also added to SOC as a salvage therapeutic option once tumors developed resistance to SOC dual therapy (palbociclib was added day 58 for Mela16 and day 21 for Mela11 and Mela14). (Figure 4A–4C). Comparing end point tumor volume, this strategy significantly inhibited tumor growth and caused tumor regression compared to single agent palbociclib with the exception of salvage therapy versus single agent palbociclib in the Mela14 model (*p* = 0.3) (Figure 4C). We additionally discovered that upfront DTP treatment provided more partial and complete regressions than any of the other treatment group as seen in Table 3 with 2/10 partial regression in Mela16 and 1/10 complete regressions in Mela14. Mela11 had 7/10 partial regressions and 2/10 complete regression. Taken together, these results show that the upfront use of triple DTP therapy is superior in delaying tumor growth and promoting tumor regression compared to dual SOC treatment and salvage therapy. The addition of palbociclib as a salvage agent to SOC therapy caused sustained tumor regression in Mela11 tumors and multiple regressions in Mela16 and Mela14 tumors as evidenced by initial tumor regressions that escaped therapy followed by additional durable regressions. Percent change in body weight provided evidence of minimal toxicity (Figure 4D–4F). Additionally, no treatment related deaths were observed.

**Table 2:** Dosing schedule of dabrafenib, trametinib, and palbocicib in combination

Treatment schedule														
Regimen 1					Regimen 2					Regimen 3				
Agent	Vehicle	mg/kg	Route	Schedule	Agent	Vehicle	mg/kg	Route	Schedule	Agent	Vehicle	mg/kg	Route	Schedule
placebo	-	-	-	-	placebo	-	-	-	-	placebo	-	-	-	-
palbociclib	-	100	po	qd	-	-	-	-	-	-	-	-	-	-
dabrafenib	-	25	po	qd	trametinib	-	1	po	qd	-	-	-	-	-
dabrafenib	-	25	po	qd	trametinib	-	1	po	qd	palbociclib	-	100	po	qd
dabrafenib	-	25	po	qd	trametinib	-	1	po	qd	palbociclib (when tumor progresses)	-	100	po	qd

Mice were treated until study completion with indicated dosing and schedules. **a,** po, oral gavage; qd, everyday

**Table 3:** *In vivo* tumor responses to triple therapy combination

	Partial regressions			Complete regressions		
	Mela16	Mela11	Mela14	Mela16	Mela11	Mela14
**placebo**	0	0	0	0	0	0
**palbociclib**	0	0	0	0	1	0
**DT**	1	0	0	0	0	0
**DTP**	2	7	0	0	2	1
**DTP sal**	0	0	0	0	0	0

A partial regression indicates that the tumor volume was 50% or less of its day one volume for three consecutive measurements during the course of the study, and equal to or greater than 13.5 mm3 for one or more of these three measurements. A complete regression indicates that the tumor volume was less than 13.5 mm3 for three consecutive measurements during the course of the study. Animals were scored only once during the study for a partial regression or complete regression event and only as complete regression if both partial regression and complete regression criteria were satisfied.

We performed additional *in vivo* drug combination studies to assess their antitumor growth activity in these PDTX models with less provocative results. Dosing schedule and treatment groups that included single agent palbociclib, single agent MK-2206 (pan-AKT inhibitor), and the combination of both drugs are described in Supplementary Table 2. We found that the combination of palbociclib and MK-2206 had similar antitumor growth activity as palbociclib alone with no combinatorial effect observed in all three models (Supplementary Figure 2A–2C). Loss in body weight provided evidence of minimal toxicity (Supplementary Figure 2D–2F). Our second investigative drug cocktail included the combination of SOC treatment with TMZ (DTT) as an upfront treatment and as a salvage therapy added to DT. The dosing schedule for these groups is described in Supplementary Table 3. Both the DTT and salvage therapies had no significant differences in response between each other and to the SOC therapy in all three models (Supplementary Figure 3A–3C). In addition to no increased treatment benefit, DTT therapy showed no evidence of body weight loss (Supplementary Figure 3D–3F). These data are shown to emphasize the profound antitumor effect of the BRAF/MEK/CDK4/6 inhibitor combination in *BRAF*^*V600E*^-mutant metastatic melanomas (Figure 4).

### Triple therapy significantly reduces cellular proliferation

**Figure 5:**
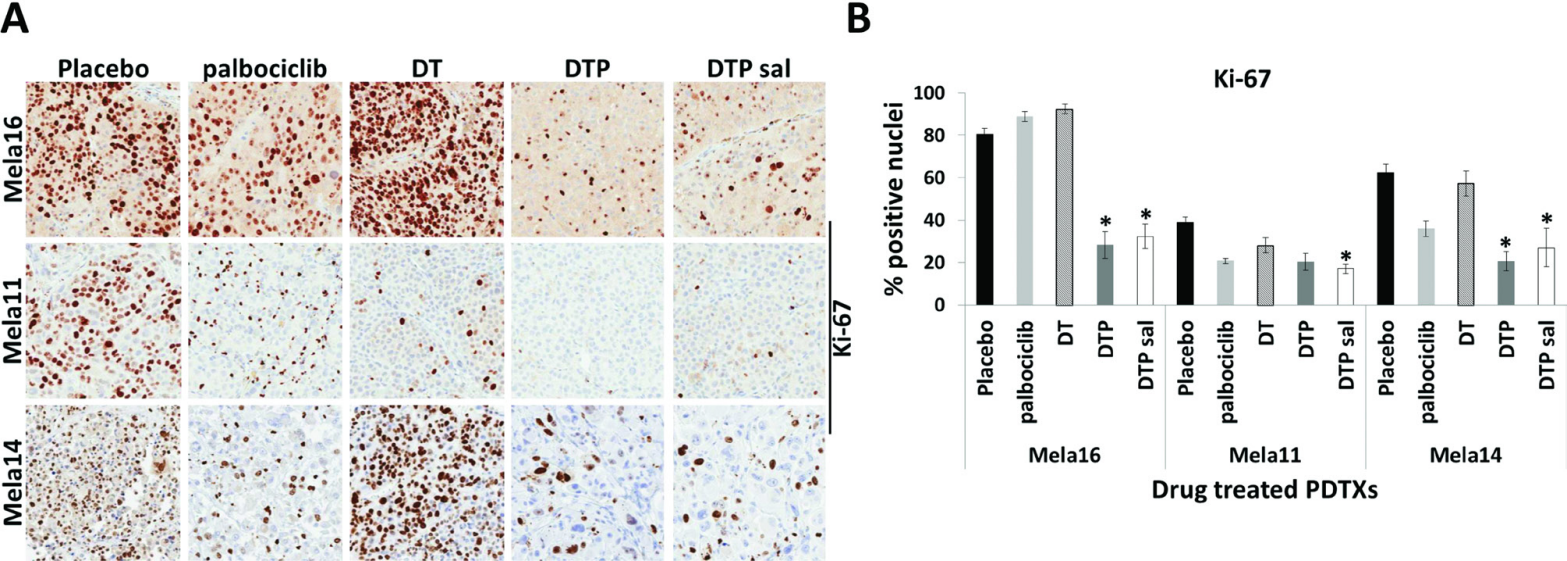
Changes in Ki-67 protein expression among treatment groups (**A**) Representative images from endpoint tumors of Ki-67 immunohistochemical staining in placebo control (*n* = 10) and therapy treated tumors (*n* = 10). Magnifications are 20×. (**B**) Quantitative analysis of Ki-67 staining. The percentage of nuclear positive tumor cells were quantitated for placebo and drug treated groups using Aperio Eslide Manager (Leica biosystems). Data are presented as the mean ± SEM. Statistical analyses are compared between DT and DTP± salvage therapy. Mean values were compared using Wilcoxon rank sum test, *p* < 0.05. a, DT, dabrafenib + trametinib; DTP, dabrafenib + trametinib + palbociclib; sal, salvage.

Since DTP therapy provided durable response compared to other combinations tested, we collected tumors from the end of the study, while still on treatment, and constructed a TMA to examine Ki-67 protein expression via IHC from each treatment group across all three models (Figure 5A). Statistical analyses from this data were performed as shown in Figure 5B using a 2-sample *t*-test. Ki-67 staining was significantly decreased in both combination groups containing palbociclib compared with vehicle control or compared to dual dabrafenib and trametinib combination (DT) with the exception of Mela11 DTP vs. DT (Figure 5B). Triple therapy combination group and salvage therapy were statistically significant in all three models when compared to SOC therapy (*p* < 0.05) with the exception of Mela11 upfront triple therapy versus SOC therapy (*p* = 0.2) (Figure 5B). These data support the notion that Ki-67 staining can distinguish triple therapy from placebo control and that triple therapy leads to decreased tumor cell proliferation with either upfront treatment or treatment in the salvage therapy setting.

### Cell signaling analysis in BRAF mutant tumors treated with dabrafenib, trametinib, and palbociclib significantly suppress pRb-Ser780

**Figure 6:**
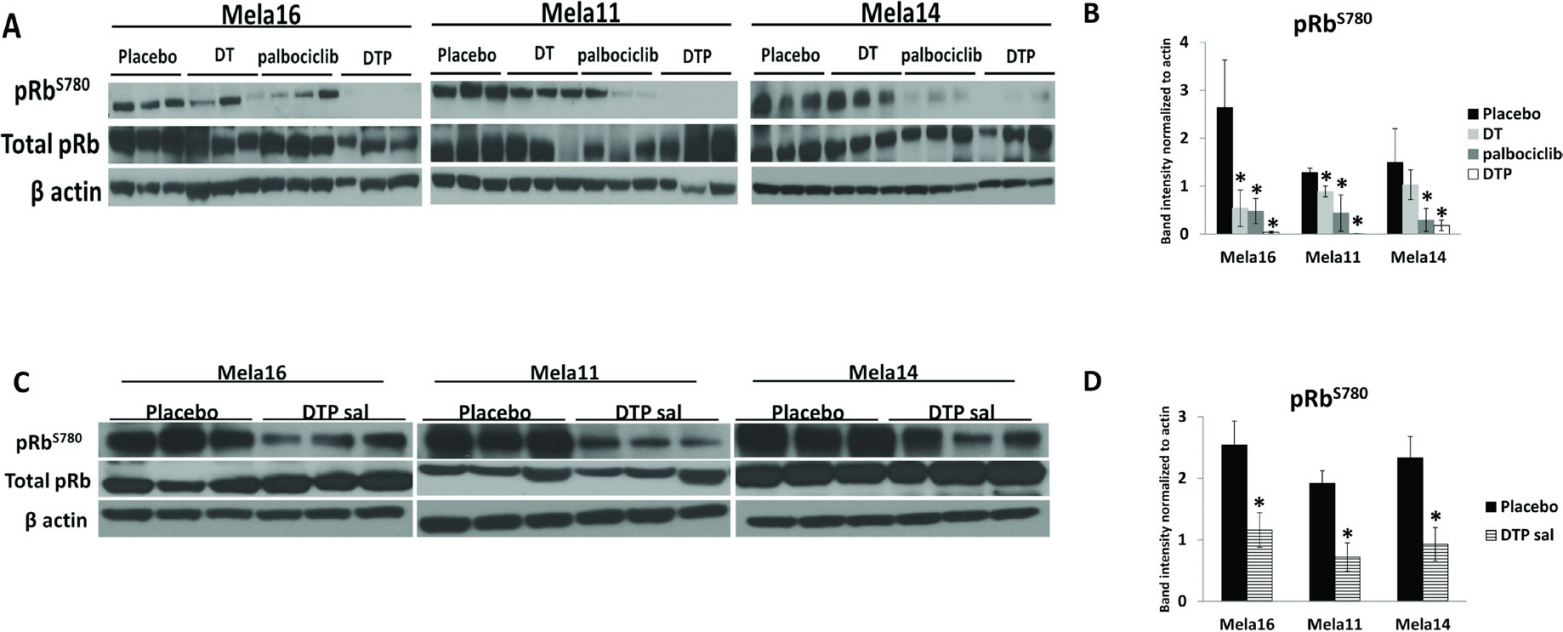
Cell signaling analysis in BRAF mutant tumors treated with dabrafenib, trametinib, and palbociclib identify pRb-Ser780 as biomarker (**A**) Ten mice were dosed, as previously described, with the indicated treatments and protein lysates were extracted from snap frozen tumors collected at the end of the study. Immunoblotting using the antibodies indicated were repeated in triplicates using all mice, representative blots are shown (*N* = 3). (**B**) pRb-Ser780 relative intensities were normalized to beta actin rather than total pRb due to variability within total pRb protein levels ± standard deviation. Statistical analysis is compared between no treatment and treated samples using a 2-sample *t*-test. The data used for this analysis were the raw mean values. (**C**) Representative images and (**D**) statistical analyses for DTP salvage therapy.

To investigate potential biomarkers indicative of response to therapy, we conducted Western blot analysis of frozen tissues taken at the time of experimental endpoint (Figure 6). pRb-Ser780 protein levels were decreased with palbociclib treatment compared to placebo and SOC treated tumors, and the addition of palbociclib to SOC therapy provided near complete suppression of pRb-Ser780 protein in all three models (Figure 6A–6B). The use of palbociclib as a salvage therapy also suppressed pRb-Ser780 protein levels (Figure 6C–6D). Collectively, these results indicate sustained activity of cell cycle signaling with SOC therapy that is significantly reduced with the administration of triple therapy. Thus, the common observation and correlate for antitumor response to triple therapy in all three models is suppression of pRb-Ser780; these results were validated in a separate set of samples collected one week after treatment (Supplementary Figure 4). Results were similar as tumors treated long-term (Figure 6). Our results show that loss of pRb-Ser780 protein expression correlates with response to therapy, suggesting that triple therapy restored pRb function, which may be associated with the decreased proliferation index found in Figure 5A. As a note, we used β-actin to normalize and quantitate pRb-Ser780 levels since total pRb was variable within each treatment group.

## DISCUSSION

We report that PDTX mouse models recapitulate tumor heterogeneity and patient response to therapy, thus providing viable preclinical tools to investigate patient-relevant therapies. In this study, we characterized three *BRAF*^*V600E*^-mutant PDTX mouse models with disparate responses to dual dabrafenib and trametinib SOC, and discovered that palbociclib sensitized tumors to SOC by eliciting tumor regressions and durable responses in BRAF/MEK inhibitor responsive and resistant tumors when used as an upfront treatment. Our data strongly support the conclusion that the addition of a CDK4/6 inhibitor to dual BRAF/MEK inhibition provides superior antitumor inhibition compared to dual BRAF/MEK inhibition or single agent palbociclib, *in vivo*.

The development of novel oncology agents is marginal compared to other disease areas [30]. Fewer than 10% of agents with preclinical antitumor activity are licensed after establishing appropriate efficacy in phase III testing [31], thus, highlighting the need for appropriate preclinical models to help reduce drug attrition rates. Since PDTX mouse models maintain human tumor heterogeneity and mimic patient response to therapy, they have been reported as respectable preclinical tools to address this issue. Several preclinical investigations report the use of melanoma PDTXs to help guide patient therapy. In a study conducted by Einarsdottir *et al.* NOD scid gamma (NSG) mice were used to develop PDTX models as an individualized approach to guide patients with stage III and IV metastatic melanoma to the right treatment [32]. One patient within this study had an objective response to treatment, as well as reductions in plasma S100β levels (a biomarker used to show response to treatment), which was recapitulated in the PDTX models. In a separate study, PDTX models were developed to facilitate individualized treatment decisions for drug resistant patients [33]. The identification of actionable drugs per tumor sample was chosen following results from whole-exome sequencing analyses. As a result, one particular sample was found to have a mutation in the *BRAF* gene, which prompted treatment with vemurafenib and its analog, PLX4720, to the patient and matched-PDTX, respectively. The PDTXs followed the same treatment response as the patient, initial response followed by drug resistance. Although we did not use our models to guide patient therapy, we did show that they mimicked patient treatment response (Figure 3). Collectively, these studies show that melanoma PDTXs correlate with patient response to therapy.

The cell cycle is regulated by the interaction between cyclins and their catalytic counterparts, CDKs. The CDK4/6/cyclin D1 complex is a key driver of G1 phase to S phase transition of the cell cycle, which leads to the phosphorylation and inactivation of the retinoblastoma protein (pRb) [34]. This interaction can be blocked by the tumor suppressor protein, p16, which promotes hypophosphorylation of the Rb protein, which inhibits cell proliferation by repressing the E2F family transcription factors and histone deacetylases [35, 36]. Thus, disruption of the Rb pathway promotes the proliferation of aberrant cells, thereby rendering them insensitive to antigrowth factors that would normally keep cells in the G1 phase of the cell cycle [37] or target cells for destruction or repair. With the knowledge that pRb is 95% wild-type in *BRAF* mutant melanoma [27] coupled with our discovery that all examined stage IV *BRAF*^*V600E*^-mutant melanoma tissues express nuclear hyperphosphorylated pRb-Ser780 reasons that blockade of the CDK4/6-cyclin D1 pathway should lead to active hypophosphorylated pRb, thus causing cell cycle arrest. Our data confirm this rationale.

pRb-Ser780 may also serve as a biomarker predicting response to CDK4/6 inhibitor therapy (Figure 1) as well as a biomarker of response not only to CDK4/6 inhibitor therapy but also triple therapy (Figure 6). Identifying and validating reliable biomarkers of response to CDK4/6 inhibitors for clinical and preclinical studies remains to be established; loss of p16 protein expression, Ki-67 index, *CCND1* amplification, and cyclin D1 protein expression have been tested in other tumor types but are not reliable predictors [38]. Currently, estrogen receptor-positive, human epidermal growth factor receptor 2 (HER2)-negative status in breast cancer patients is the only used predictive biomarker for response to CDK4/6 inhibition [39, 40]. While investigating the antitumor activity of our triple therapy combination, we observed that upfront triple therapy was the only combination to elicit both partial and complete tumor regressions in our PDTX models. This most likely is due to blockade of cell cycle progression leading to attenuation of cellular proliferation as evidenced by decreased Ki-67 protein expression (Figure 5) and pRb-Ser780 protein levels (Figure 6). Our results validate findings similar to human metastatic melanoma patient samples that developed acquired resistance to BRAF inhibition [23]. In a study by Trunzer *et al.* 22 paired biopsies from patients with metastatic melanoma were examined to elucidate mechanisms of intrinsic and acquired resistance to single agent vemurafenib. In their study, treatment naïve tumors with high Ki-67 protein expression were decreased with daily dosing of vemurafenib. Once tumors became resistant to therapy, protein levels were elevated [23]. In our study, we found that Ki-67 protein expression remained decreased when palbociclib was combined with BRAF/MEK inhibitors (Figure 5). Additionally, our data suggests that palbociclib sensitized tumors to SOC treatment, shown by partial and sustained tumor regressions with salvage therapy, durable regression with upfront therapy, and decreased pRb-Ser780 (Figure 4 and Figure 6). This most likely is a consequence of the restored function of pRb to its dephosphorylated state. Similar results indicating the role of CDK4/6 inhibition in melanoma cell cycle signaling were observed in a study by Yadav *et al.* who showed that abemaciclib (CDK4/6 inhibitor) caused significant tumor regression in a single xenograft mouse model developed from vemurafenib resistant A375 cell lines [19]. As previously mentioned, there are many mechanisms of resistance to BRAF/MEK inhibition, each of which can vary between and within melanoma tumors. Because of this, we additionally investigated protein expression levels of cyclin D1, pERK, and CDK4 as potential mechanisms of resistance to SOC in our PDTX mouse models; all of these proteins provided inconclusive results (data not shown). However, our clinically relevant preclinical PDTX mouse models confirm and support the conclusion that BRAF/MEK inhibitors combined with CDK4/6 inhibitors effectively inhibits tumor growth in *BRAF*^*V600E*^-mutant melanomas by simultaneous or subsequent targeting of the cell cycle machinery.

Preclinically, there are conflicting results on how best to administer CDK4/6 inhibitors to maintain efficacy and reduce toxicity. Results from a proliferation and colony forming assay demonstrated that sequential and intermittent treatment with BRAF inhibition and palbociclib was not as effective as continuous combination dosing [41]. Alternatively, utilizing human liposarcoma cell lines and PDTX mouse models, Zhang *et al.* demonstrated that continuous chronic exposure to single agent ribociclib (CDK4/6 inhibitor) led to reversion of RB hyperphosphorylation at the CDK4/6-specific sites S780 and S807/811 [42], suggesting that scheduled intermittent dosing may be more beneficial in maintaining cell cycle arrest. This phenomenon has also been reported in a subset of estrogen receptor positive breast cancer cell lines [43]. However, in our experiments, after weeks of continuous treatment with palbociclib ± SOC therapy (Figure 4A–4C), we saw minimal reversion of pRb-Ser780 with single agent palbociclib treated tumors; pRb-Ser780 levels were consistently and significantly suppressed with triple therapy treated tumors at the protein level (Figure 6). Moreover, results from our study indicate that rather than discontinuing SOC therapy, palbociclib should be added to SOC therapy as a triple therapy approach in naïve and SOC drug resistant *BRAF*^*V600E*^-mutant melanoma.

During our investigation of the combinatorial antitumor effects of BRAF, MEK, and CDK4/6 inhibition in *BRAF*^*V600E*^*-*mutant PDTX melanoma models, results from a phase Ib/II clinical trial evaluating the same drug class combination in treatment naïve *BRAF*^*V600-*^*-*mutant solid tumors, including melanoma, were recently presented at the 2017 American Society of Clinical Oncology (ASCO) meeting [44]. Provisional results from this study reported an objective response rate of 52.4% with four complete responses, eighteen partial responses, and fifteen cases of stable disease. Despite the robust antitumor activity reported in our study and this clinical trial, we cannot negate the fact that there are concerns related to drug toxicities using triple therapeutic agents. While we observed minimal decreases in body weight as an indicator of drug toxicity (Figure 4D–4F), CDK4/6 inhibitors demonstrated clinical toxicities requiring careful management in patients. Evidence of increased toxicity (i.e. including neutropenia, increased alanine transaminase, diarrhea, and anemia) in the ASCO trial was reported with 24% of patients discontinuing treatment, confirming that dose-limiting toxicity of myelosuppression is consistent with the on-target inhibition of CDK4/6 inhibitors [45]. The use of a third CDK4/6 inhibitor (abemaciclib) is reported to have a lower toxicity profile than palbociclib and ribociclib as evidenced by decreased myelosuppression [46]. This CDK4/6 inhibitor was recently approved for (HR)-positive, HER2-negative breast cancer patients that have progressed on endocrine therapy. A recent study by Yoshida *et al.* reported that short-term treatment (8 days) with palbociclib induced senescence in vemurafenib resistant melanomas [27]. The translational clinical relevance remains to be investigated but if clinically proven, reduced treatment of CDK4/6 inhibition following acquired drug resistance to BRAF/MEK inhibition could minimize toxicity profiles associated with CDK4/6 inhibition through permanent growth arrest.

The initial clinical impact of BRAF inhibitors is significant in *BRAF*^*V600E*^-positive tumors; however, long-term treatment benefits are often limited due to rapidly acquired drug resistance. Hence, the development of secondary treatment strategies for drug resistant tumors is of great importance. Despite recent successes in antitumor growth activity (including our study) with BRAF/MEK/CDK4/6 inhibition in *BRAF*^*V600E*^*-*mutant melanomas [44], some may argue against using the triple therapeutic agent following recent reports of sustained clinical OS and progression-free survival (PFS) rates lasting up to five years in a proportion of melanoma patients [47, 48]. Since the initial study of BRAF/MEK inhibition in *BRAF*^*V600-*^ mutant tumors [49], PFS has significantly improved [50]. Long *et al.* recently conducted a five year landmark clinical study on the prolonged SOC treatment benefit in histologically confirmed unresectable stage IIIC or IV *BRAF*^*V600E/K*^ -mutant melanoma patients. In this study, OS rates of 30% and 28% were associated with four and five year survival, respectively. PFS for both years was 13% [48]. Even with these successes, it is evident that a select population of patients received continued benefit to SOC therapy. Specifically for patients who received 5 year treatment benefit, increased OS was associated with those who had favorable baseline factors [i.e. normal lactate dehydrogenase baseline levels (45%) and involvement of less than three organ sites with metastases (51%)] [48]. Although differences in response rates between triple BRAF/MEK/CDK4/6 inhibition (52.4%) and dual BRAF/MEK inhibition (63%) have been reported [44, 51], the long-term therapeutic benefits and intrinsic or acquired resistance of BRAF/MEK/CDK4/6 inhibition are currently unknown. Importantly, as shown in our study, BRAF/MEK/CDK4/6 inhibition produced sustained and durable response in *BRAF*^*V600E*^-mutant melanoma patient-derived preclinical models regardless of drug sensitivity. These results may translate clinically by significantly delaying or preventing acquired drug resistance which commonly occurs with SOC therapy. Additionally, triple therapy treatment may increase survival benefits in patients with less favorable baseline factors, thereby improving the percent of patients who survive long-term. Since palbociclib inhibits the cytotoxic activity of BRAF inhibition [27], sequencing strategies of these therapies will be crucial.

To conclude, we investigated the antitumor effect of a triple therapy combination in PDTX mouse models derived from human metastatic melanomas. These preclinical models recapitulated human tumor heterogeneity and patient response to treatment, confirming their ability to provide insight into clinically relevant novel therapies. In all PDTX models tested, the combination of upfront palbociclib with SOC provided superior response characterized by tumor regression and durable treatment. Additionally, when palbociclib was used as a salvage therapy multiple and sustained tumor regressions were achieved. Although the molecular mechanism whereby inhibition of CDK4/6 inhibition impacts BRAF/MEK inhibition remains to be further investigated, we observed that simultaneous and subsequent treatment of palbociclib and SOC therapy in our *BRAF*^*V600E*^-mutant PDTXs significantly sustained tumor growth inhibition superior to SOC or single agent palbociclib . Our studies further showed that triple therapy combination restored the activity of pRb and decreased cellular proliferation thus blocking cell cycle progression. Therefore, we predict that sustained inhibition of phosphorylated Rb-Ser780 is a contributor of continued tumor growth inhibition *in vivo*. This finding may indicate suppressed phosphorylated Rb-Ser780 as a reliable predictive biomarker for response to therapy; however, studies in larger sample sizes are necessary. Thus, our data provide the foundation to investigate the presence and loss of pRb-Ser780 as a biomarker of acquired resistance to BRAF/MEK inhibition and a biomarker of response to BRAF/MEK/CDK4/6 therapy in BRAF^*V600E*^-mutant melanomas, respectively. As noted, while our studies were in progress, clinical trial data confirmed strong responsiveness to the triple therapy but also a cautionary note of excessive toxicity. Our data importantly showed sustained response as well as resensitization of SOC drug resistant tumors. It will be of great interest to follow the results of this trial over time to see if our preclinical model results are born out in this trial and certainly other trials testing this triple therapy.

## MATERIALS AND METHODS

### Development of PDTX mouse models

Biopsy or surgically resected tumor tissues from metastatic melanomas were implanted subcutaneously into 6 to 8 week old anesthetized athymic nude female mice strain #069 (Harlan Laboratories, Indianapolis, IN) under IACUC approved procedures. Implanted tumors were harvested and cryogenically frozen as 5 mm^3^ fragments in 10% DMSO-DMEM media. Serial mouse-to-mouse passaging was continued to create a renewable source of metastatic melanoma tissue and representative *in vivo* models to test promising drugs. These melanoma PDTX mouse models were deposited at Charles River Laboratories, Inc. (Morrisville, NC) and are identified as ME-022 (Mela11), ME-016 (Mela14), and ME-023 (Mela16). In this study, tumor passages 6, 5, and 6 were used for Mela11, Mela14 and Mela16, respectively.

### Patient tissues

Patient tumor tissues were collected in accordance with Mayo Clinic institutional review board protocol. Patients with biopsy proven cutaneous metastatic melanoma were identified in the pathology information system. The clinical records were reviewed to determine the AJCC stage of their disease as well as availability of clinical data and follow up information. Selection for inclusion in the tissue microarrays (TMAs) were based on the amount of tissue in the formalin-fixed, paraffin-embedded (FFPE) blocks, the availability of follow up information and the patient was classified as AJCC stage IV. Only metastatic tumors were used. Tissue microarrays were created using a Galileo Tissue Microarrayer (Integrated Systems Engineering, Philadelphia). Tissue was obtained from 203 patients and control tissues included liver, placenta, tonsil, and skin. 1 mm cores were used for the microarrays. An additional TMA of human cutaneous melanoma tumors was constructed along with matched tumors from PDTXs.

### Immunohistochemistry and hematoxylin and eosin (H&E) stains

All FFPE tissues and TMAs were cut into 5μm sections, deparaffinized, hydrated, antigen retrieved and blocked with Diluent that contained Background Reducing Components (Dakocytomation, Denmark). Immunostaining was done on either the TMA or single section tissues alone with the following: human lamin A+C [1:400, anti-rabbit (Novus Biologicals, Littleton, CO)]; pERK [1:100, anti-rabbit (Cell Signaling, Beverly, MA)]; Rb-phospho S780 [1:50-1:100, anti-rabbit (Abcam, Cambridge, MA)]; Ki-67 [1:100, anti-rabbit (Novus Biologicals, Littleton, CO)]; CDK4 [1:100, anti-rabbit (Cell Signaling, Beverly, MA)]; and CDK6 [dilution, anti-rabbit (Cell Signaling, Beverly, MA)]. Images were obtained using Scanscope XT (Aperio Technologies, Vista, CA) and algorithms generated in the Imagescope software (Aperio Technologies) were used to score the TMA punches. All cases of insufficient tumor tissue were excluded. 20x images were obtained using Scanscope XT and Imagescope software. This study was approved by the Mayo Clinic Institutional Review Board.

### Efficacy studies in PDTX mouse models

All animal experiments were done according to an Institutional Animal Care and Use Committee–approved protocol. Institutional guidelines for the proper and humane use of animals in research were followed. Animals were used between the ages of 8 to 12 weeks (Charles River Discovery, Morrisville, North Carolina). Human tumor fragments (5 mm^3^) were suspended in 50% PBS and were injected subcutaneously into mouse flanks using a trocar. Mice were randomized into control and treatment groups when average tumor size reached 100–150 mm^3^ (*n* = 10 animals per group). The following vehicles were used to dose the compounds: 30% Captisol (Cydex) for MK-2206 (AKT inhibitor; Selleckchem); 0.5% hydroxypropyl methylcellulose + 0.2% Tween 80 in distilled water for dabrafenib (BRAF inhibitor; LC laboratories) and trametinib (MEK inhibitor; LC laboratories); 50 mM sodium lactate buffer for palbociclib (CDK4/6 inhibitor; LC laboratories); distilled water for temozolomide (alkylating agent; Merck and Co.); saline for bevacizumab (VEGF inhibitor; Genentech); saline for abraxane (mitotic inhibitor; Abraxis); and saline for cisplatin (alkylating agent; TEVA) . The control group received vehicle only. Tumor volumes were measured with calipers as indicated by treatment schedule. Animal body weight and physical signs were monitored during the experiments. Tumor volume was calculated, taking length to be the longest diameter across the tumor and width to be the perpendicular diameter, by using the following formula: (length × width) ^2^ × 0.5.

### DNA isolation and short tandem repeat (STR) analysis

Genomic DNA from primary tissues and matching cell lines and PDTX mouse tissues were isolated using the Purelink Genomic DNA mini kit (Invitrogen, Carlsbad, CA). These samples were amplified and analyzed against twelve STR markers as previously described [52] by the Mayo Clinic Medical Genome Facility Genotyping Core.

### Preparation of cell lysates and immunoblotting

Protein extraction and Western blot analysis for cells were performed as previously described [53]. Frozen tumor tissues were homogenized and lysed in 5 volumes cold RIPA lysis buffer (Pierce), containing 1x phosphatase (Thermo Scientific) and protease inhibitor cocktail (Roche). Primary antibodies included: phospho-p42/p44 extracellular signal–regulated kinase (ERK) [1:1000, anti-rabbit (Cell Signaling, 4376)], total ERK [1:1000, anti-rabbit (Cell Signaling, 9102)], CDK4 [1:1000, anti-rabbit (Cell signaling, 12790)], CDK6 [1:1000, anti-rabbit (Cell signaling, 12790)], cyclin D1 [1:2000, anti-mouse (Cell Signaling, 2926)], p-S780 Rb [1:500, anti-rabbit (Abcam, ab44763)], total RB [(1:200, anti-rabbit (Santa Cruz, sc-50)], and beta actin [(1:5000, anti-mouse (Sigma, A5441). The protein-antibody complexes were detected by using an enhanced chemiluminescence kit (ThermoFisher Scientific) according to the manufacturer’s recommended protocol.

### Statistical analysis

For *in vivo* studies statistical significance was evaluated using the Wilcoxon rank test comparing novel combination treatments to vehicle, single agent therapy, dabrafenib plus trametinib, or salvage therapy. A 2-sample *t*-test was used to determine statistical significance for immunohistochemistry and Western blot analysis.

## SUPPLEMENTARY MATERIALS FIGURES AND TABLES



## Abbreviations

(CDK4/6): cyclin dependent kinase 4/6
(CDK): cyclin dependent kinases
(DT): dabrafenib + trametinib
(DTP): dabrafenib + trametinib + palbociclib
(DTT): dabrafenib + trametinib + temozolomide
(IHC): immunohistochemistry
(MAPK): mitogen-activated protein kinase
(PDTX): patient-derived tumor xenograft
(Rb): retinoblastoma
(pRb): retinoblastoma protein
(STR): short tandem repeat
(SOC): standard of care
(TMZ): temozolomide
(TMA): tissue microarray
(UVR): ultraviolet radiation
