# Supplementary material for: Targeting the cyclin dependent kinase and retinoblastoma axis overcomes standard of care resistance in BRAFV600E-mutant melanoma
Source: Oncotarget. 2017 Dec 23;9(13):10905–19. doi: 10.18632/oncotarget.23649 (PMC5834273; doi:10.18632/oncotarget.23649)
Supplement: Supplementary file 1 [file oncotarget-09-10905-s001.pdf]

Targeting the cyclin dependent kinase and retinoblastoma axis overcomes standard of care resistance in BRAF^{V600E}-mutant melanoma

SUPPLEMENTARY MATERIALS

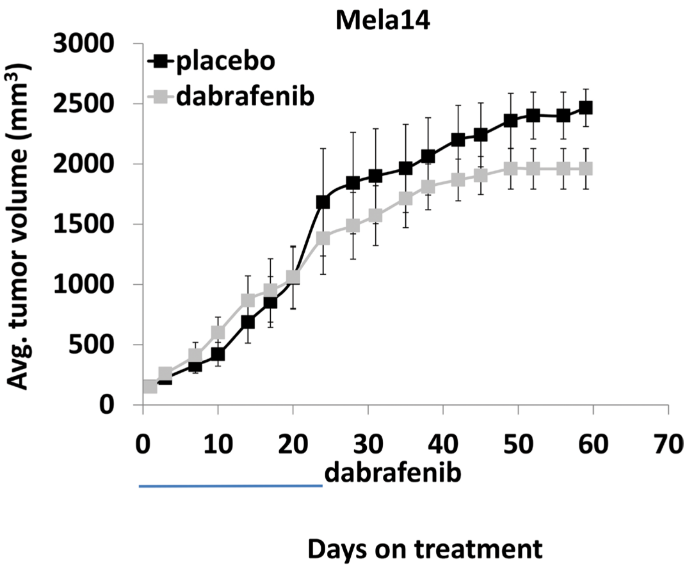

Supplementary Figure 1: Tumors escape drug inhibition after drug cessation. (A) Athymic nude mice bearing subcutaneous tumors (n = 8) were dosed with vehicle or dabrafenib (25 mg/kg) as indicated. Using the Wilcoxon rank sum test comparing placebo to dabrafenib treatment revealed no statistical significance between groups on tumor growth, $p = 0.43$. The blue line below the x-axis indicates dosing (Rx) period in all studies. The y-axis is mean tumor volume ± SEM.

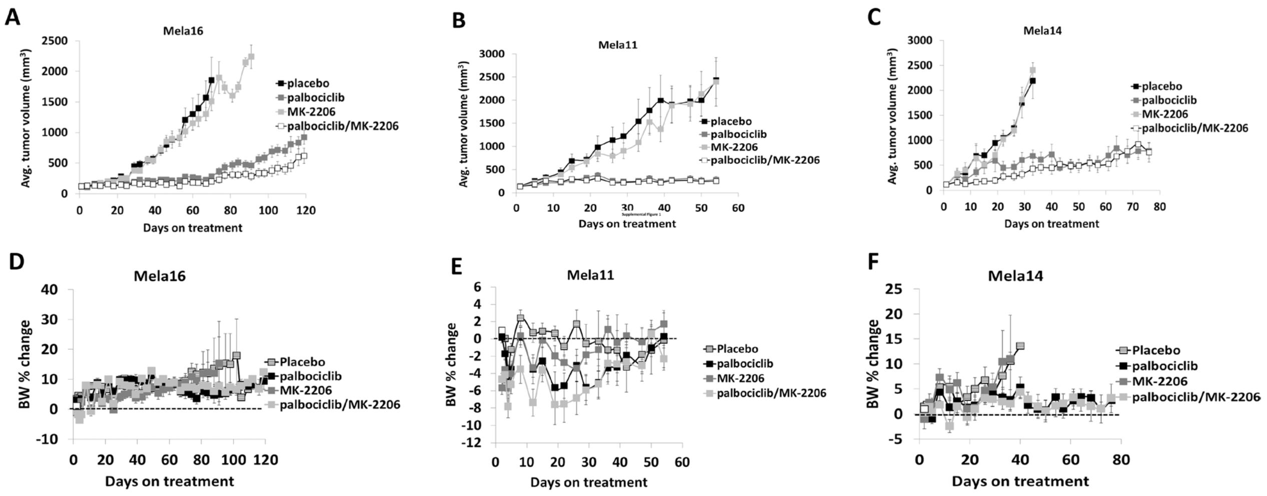

Supplementary Figure 2: Single agent palbociclib is as effective as dual combination with MK-2206. (**A–C**) Athymic nude mice bearing subcutaneous tumors were dosed time to endpoint in well-established tumors (~150 mm^3). The y-axis is mean tumor volume ± SEM. With the exception of vehicle versus MK-2206 in all PDTX models ($p = 0.9$, Mela11; $p = 0.48$, Mela14; $p = 0.71$, Mela16), pairwise comparisons of each treatment versus control are all statistically significant ($p < 0.05$). Tests between the combination groups and both single agent therapies are also statistically significant ($p < 0.05$), with the exception of palbociclib versus palbociclib and MK-2206 combination ($p = 0.9$, Mela11; $p = 1$, Mela14). Statistical analyses between palbociclib and MK-2206 are also statistically significant in all groups ($p < 0.01$). (**D–F**) Change in percent body weight loss was calculated per mouse. The y-axis is change in body weight measured from baseline (day 1) ± SEM.

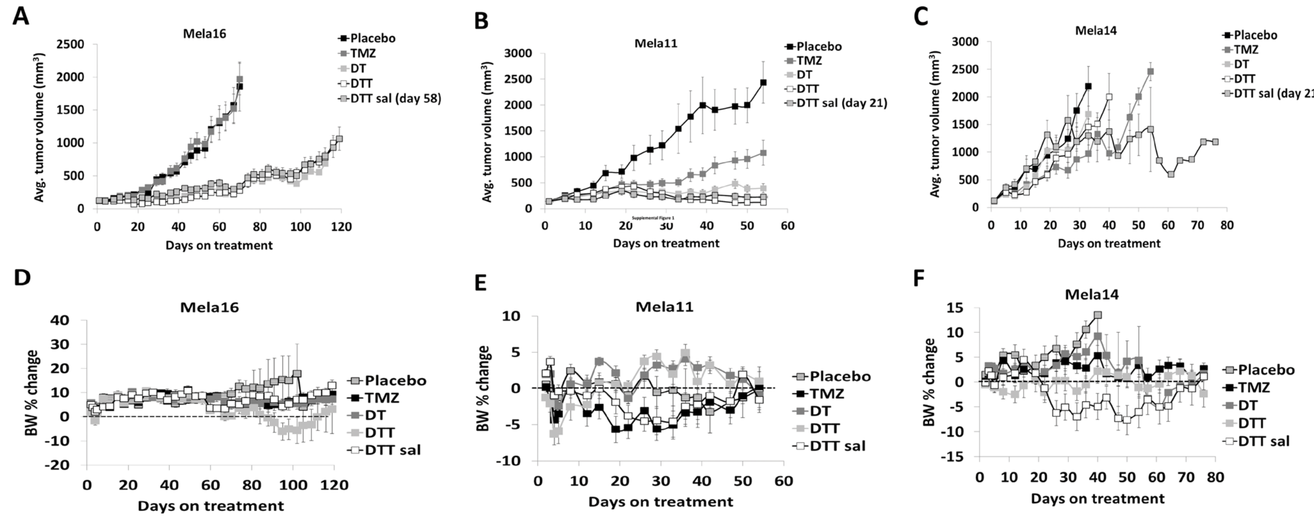

Supplementary Figure 3: The addition of temozolomide to dabrafenib and trametinib provides to no treatment benefit to standard of care therapy *in vivo*. (**A–C**) Athymic nude mice bearing subcutaneous tumors were dosed time to endpoint in well-established tumors (~150 mm^3). The y-axis is mean tumor volume ± SEM. With the exception of Mela14 (placebo versus TMZ, $p = 0.72$; placebo versus dabrafenib and trametinib, $p = 0.46$; placebo versus dabrafenib, trametinib, and TMZ, $p = 0.92$ placebo versus dabrafenib, trametinib, and TMZ salvage, $p = 0.29$), pairwise comparisons of each treatment versus control are all statistically significant ($p < 0.05$). Tests between the combination groups versus dabrafenib and trametinib combination were not statistically significant (dabrafenib and trametinib versus TMZ, $p = 0.92$, Mela14; dabrafenib and trametinib versus dabrafenib, trametinib, and TMZ, $p = 0.10$, $p = 0.58$, $p = 0.09$ Mela11, Mela14, Mela16, respectively; and dabrafenib and trametinib versus dabrafenib, trametinib, and TMZ salvage, $p = 0.93$, $p = 0.80$, $p = 0.07$ Mela11, Mela14, Mela16, respectively), with the exception of dabrafenib and trametinib versus TMZ ($p < 0.05$) for both Mela11 and Mela16. With the exception of Mela14 (TMZ versus DTT, $p = 0.52$; TMZ versus DTT salvage, $p = 0.37$), pairwise comparisons of DTT treatment ± salvage versus TMZ were all statistically significant ($p < 0.05$). Statistical analyses between DTT and DTT salvage were not statistically significant ($p = 0.05$, $p = 0.49$, $p = 0.6$ Mela11, Mela14, Mela16, respectively). (D–F) Change in percent body weight loss was calculated per mouse. The y-axis is change in body weight measured from baseline (day 1) ± SEM.

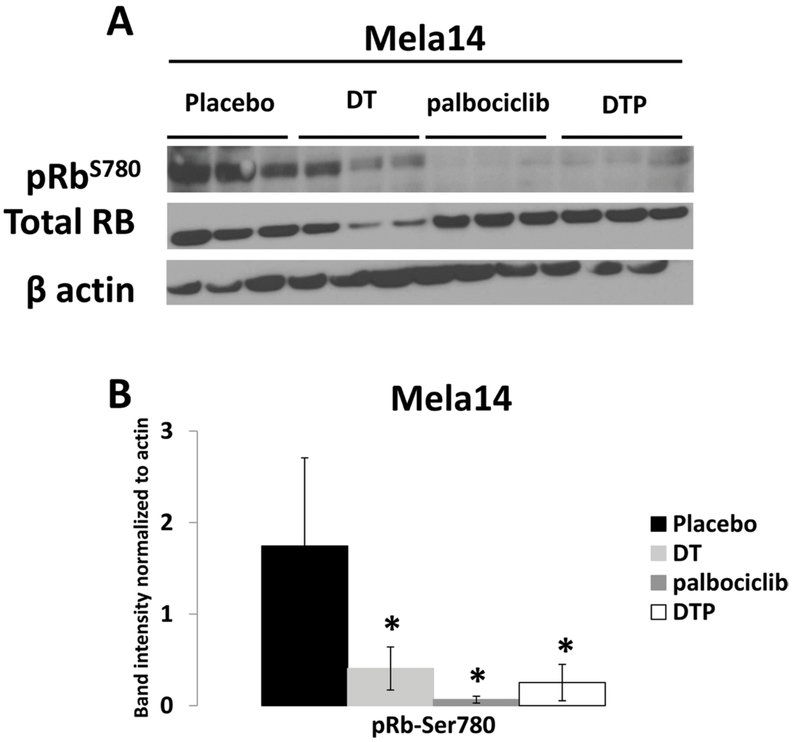

Supplementary Figure 4: Early treatment effects of triple therapy indicate pRb as a biomarker for response to therapy. (**A**) Six mice were dosed, as previously described, with the indicated treatments for one week and protein lysates were extracted from snap frozen tumors collected after treatment. Immunoblotting using the antibodies indicated were repeated in triplicates using all mice, representative blots are shown. (**B**) Graphical representation of the relative intensities of the indicated antibodies normalized to beta actin control ± standard deviation. Statistically analysis is compared between no treatment and treated samples using a 2-sample *t*-test. The data used for this analysis were the raw mean values.

Supplementary Table 1: Synergistic indexes of combination treatment with dabrafenib, trametinib, and palbociclib *in vivo*

Xenografts	dabrafenib/trametinib		palbociclib		dabrafenib/trametinib/palbociclib			Index[D]
	MGI[A]	*p*-value[E]	MGI[A]	*p*-value[E]	Expected[B]	Observed[C]	*p*-value[E]	
**Mela16**	0.73	$p < 0.05$	0.71	$p = 0.41$	0.52	0.22	$p < 0.05$	2.36
**Mela11**	0.28	$p < 0.05$	0.23	$p = 0.17$	0.06	0.08	$p < 0.05$	0.81
**Mela14**	0.53	$p = 0.46$	0.73	$p = 0.14$	0.39	0.39	$p < 0.05$	0.99

Growth inhibition rates were calculated at the end of the experiment on BRAF mutant PDTX tumor nodules in athymic nude mice treated with dabrafenib/trametinib, palbociclib, or all three in combination. a, Mean growth inhibition rate = growth rate of treated group/growth rate of untreated group b, Growth inhibition rate of dabrafenib/trametinib × growth rate of palbociclib. c, Growth inhibition rate of combined treatment on dabrafenib/trametinib and palbociclib treatments. d, Calculated by dividing the expected growth inhibition rate by the observed growth inhibition rate. An index >1.1 indicates synergistic effect, between 0.9 and 1.1 indicates additive effect, and <0.9 indicated less than additive effect. e, *p*-values were calculated using the Wilcoxon rank sum test, where palbociclib and triple therapy are both compared to dabrafenib/trametinib combination while the dabrafenib/trametinib combination is compare to control treatment. MGI, mean growth inhibition.

Supplementary Table 2: Dosing schedule of palbocicib and MK-2206 in combination

Regimen 1					Regimen 2				
**Agent**	**Vehicle**	**mg/kg**	**Route**	**Schedule**	**Agent**	**Vehicle**	**mg/kg**	**Route**	**Schedule**
placebo	-	-	-	-	placebo	-	-	-	-
palbociclib	-	100	po	qd	-	-	-	-	-
MK-2206	-	450	po	qwk	-	-	-	-	-
palbociclib	-	100	po	qd	MK-2206	-	450	po	qwk

Mice were treated until study completion with indicated dosing and schedules. a, po, oral gavage; b, qd, everyday.

Supplementary Table 3: Dosing schedule of dabrafenib, trametinib, and temozolomide in combination

Treatment schedule														
**Regimen 1**					**Regimen 2**					**Regimen 3**				
**Agent**	**Vehicle**	**mg/kg**	**Route**	**Schedule**	**Agent**	**Vehicle**	**mg/kg**	**Route**	**Schedule**	**Agent**	**Vehicle**	**mg/kg**	**Route**	**Schedule**
placebo	-	-	-	-	placebo	-	-	-	-	placebo	-	-	-	-
TMZ	-	100	po	qd	-	-	-	-	-	-	-	-	-	-
dabrafenib	-	25	po	qd	trametinib	-	1	po	qd	-	-	-	-	-
dabrafenib	-	25	po	qd	trametinib	-	1	po	qd	TMZ	-	100	po	qd
dabrafenib	-	25	po	qd	trametinib	-	1	po	qd	TMZ (when tumor progresses)	-	100	po	qd

Mice were treated until study completion with indicated dosing and schedules. a, po, oral gavage; b, qd, everyday.
